# Molecular assemblies on surfaces: towards physical and electronic decoupling of organic molecules

**DOI:** 10.3762/bjnano.12.71

**Published:** 2021-08-23

**Authors:** Sabine Maier, Meike Stöhr

**Affiliations:** 1 Department of Physics, Friedrich-Alexander-Universität Erlangen-Nürnberg, Erwin-Rommel-Str. 1, 91058 Erlangen, Germany; 2 Zernike Institute for Advanced Materials, University of Groningen, Nijenborgh 4, 9747 AG Groningen, Netherlands

**Keywords:** decoupling layer, molecular self-assembly, scanning probe microscopy, surface science

Over the past two decades, organic molecules adsorbed on atomically defined metal surfaces have been intensively studied to obtain an in-depth understanding of their self-assembly behavior, on-surface reactivity, as well as their structural and electronic properties [1–6]. An important aspect to unravel their potential use in electronic and optoelectronic devices is how their functionality can be preserved when adsorbed on surfaces. Unfortunately, the (strong) interaction of the molecules with the metallic surface, for example, due to hybridization of molecular states with electronic bands from the metallic substrate, often alters the electronic properties of the molecules and, moreover, can even turn off their sought-after functionality. As a result of the (strong) interaction, the molecular scaffolds can also become distorted, electronic states may be significantly broadened and shifted, and vibronic states may even be quenched. Decoupling strategies offer unique opportunities to reduce these (strong) interactions. In the following, recent progress to decouple both single molecules and molecular assemblies physically and electronically from a (strongly) interacting support is briefly reviewed.

The molecule–substrate decoupling can either be achieved by suitable molecular design or manipulation of the molecular building blocks, or passivation of the substrate. Molecular engineering approaches include rigid spacers added to the molecular core [7–10] or specially designed double-decker molecules [11] to maintain the active part of the molecule sufficiently high above the surface to prevent it from interacting with the latter. Alternatively, molecular/oligomeric structures can be attached to the tip of a scanning probe microscope and mechanically lifted from the metallic surface such that they hang freely between metal contacts. This manipulation technique allows for measuring, amongst others, the electronic conductance, magnetic properties, reversible switching, and electroluminescence of free-standing molecules [12–16]. Physical decoupling strategies involving the design or manipulation of the molecular building block significantly limit the selection of building blocks and often hinder probing molecular properties with intramolecular resolution. Therefore, modifications of the substrate, for instance by adding or intercalating a decoupling layer, are often the better choice. In the best case, these interfacial layers have a large bandgap to prevent a hybridization with molecular states as well as with the metallic/semiconducting substrate. All the strategies for physical and electronic decoupling have been developed in view of fundamental studies as well as application in devices.

Ultrathin semiconducting or insulating decoupling layers can be epitaxially grown as mono- and multilayers on many metallic substrates by either physical or chemical vapor deposition. Among others, ultrathin dielectric layers of either alkali halides (e.g., NaCl [17]) or metal oxides (e.g., MgO [18], Al_2_O_3_ [19], and CuO [20]), or nitrides (CuN [21]) have been shown to be beneficial for successfully reducing or even completely switching off the unwanted interaction between the metal substrate and the organic building blocks. Recently, two-dimensional (2D) materials, including hexagonal boron nitride (*h*BN) [22–23], graphene [24–27], and MoS_2_ [28], have emerged as monatomically thin decoupling layers. Van der Waals 2D materials are generally well suited due to their chemical inertness and the low density of states near the Fermi level. However, the electronic decoupling efficiency also depends on the electronic structure of the 2D material. Sometimes, only molecular states in the bandgap of the 2D material can be decoupled. Moreover, ultrathin organic spacer layers can efficiently electronically decouple further organic layers from a metal surface. The decoupling strategy with organic layers relies on both an increased separation between the organic layer of interest and the metal as well as the different electronic gaps of the organic interface layer and following ones [29–32]. Additional concepts to weaken adsorbate–surface interactions involve the post-deposition intercalation of atomic species such as iodine [33].

For semiconductors, for example, bare silicon or germanium, electronic decoupling of molecules can be achieved by either the growth of ultrathin dielectric layers on top of the surface [34–35] or a chemical modification of the surface to saturate the dangling bonds. In surface-science-based studies, for the latter approach hydrogenation of semiconductor surfaces is frequently applied as effective passivation against chemisorption of adsorbates [36–39], while also B deposition was shown to result in effective passivation of the Si surface [40–41]. In particular for electronic devices, oxidized semiconductor surfaces (e.g., silicon dioxide layers formed on bare silicon) are mostly used as substrates for fabricating devices [42].

Most of these ultrathin interfacial layers significantly reduce both the molecular adsorption energy and the hybridization of molecular states with the electronic bands in metals and semiconductors. However, charge transport is not completely inhibited, and electrons can still tunnel from the metal through these decoupling layers to the organic molecules and vice versa. This has the great advantage that these systems can still be examined by scanning tunneling microscopy (STM) and spectroscopy (STS), which gives insight into structural and electronic properties of individual molecules. For applications such vertical tunneling through the interfacial layers can be undesirable since it prevents the current from flowing across electrode–molecule–electrode junctions [43] or leads to the charging of physisorbed molecules on top of such layers [18]. Hence, ultrathin insulating layers are often not sufficient to truly electronically insulate molecular structures. For this, the usage of either thicker insulating films (mostly alkali halides) or bulk insulators is required.

For studying molecules on bulk insulators, ionic crystals (e.g., KBr, NaCl, CaF_2_, and calcite) have mostly served as model systems. To a lesser extent, metal oxides have also been used, for which defects and charging often pose additional challenges [44–46]. On electronically insulating surfaces, non-contact atomic force microscopy (AFM) is the method of choice to study molecular assemblies and individual molecules in real space. Molecular adsorption and self-assembly are significantly altered compared to metals due to an often weak interaction between organics and bulk insulators. In contrast to face-on adsorption on metals, a tilted and edge-on adsorption becomes possible for planar aromatic molecules on bulk insulators. Due to lowered diffusion barrier and adsorption energy, the two-dimensional molecular layers can be affected by dewetting and may change into three-dimensional clusters [47]. In return, the reduced molecule–surface interaction on insulating films or bulk insulators can stabilize highly reactive molecules, offering unique opportunities for the bottom-up assembly of novel carbon-based materials using on-surface chemistry [48–49]. However, the significantly reduced catalytic activity on non-metallic substrates requires exploring alternative reaction mechanisms beyond thermal activation, for example, photon-induced reactions [46,49–51] or electron-induced reactions by electrons from a probe tip [52–53].

In the following, we would like to highlight a few examples for physical properties and built-in functionalities of molecular systems, which were successfully accessed by employing one of the decoupling strategies mentioned above. Electronic decoupling significantly increases the lifetime of excited molecular states and improves the effective energy resolution (down to a few millielectronvolts) of molecular resonances observed in tunneling spectroscopy since the hybridization of molecular states with the ones of the metallic/semiconducting support is prevented. Thereby, it became feasible to investigate molecular orbitals [17], observe and switch the charge state of individual molecules [54–59], and resolve individual vibronic states in single molecules and molecular assemblies [23,28,60–61]. Similarly, the lack of electronic states around the Fermi level in a superconductor was used to preserve electronic properties in adsorbed molecules. For example, the spin relaxation in magnetic molecules was suppressed on a superconducting surface, which then resulted in a significant enhancement of the excited-state spin lifetimes [62]. Concerning optical properties, successful decoupling made the examination of fluorescence from both single molecules and molecular assemblies feasible by tunneling electron excitation [19,63–66]. Also, sub-molecularly resolved Raman images were successfully demonstrated [67]. Additionally, electroluminescence was reported for suspended molecular wires between a metallic surface and the tip of a scanning tunneling microscope [14]. Moreover, the decoupling brings considerable benefits for surface-supported molecular switches, in particular for preserving reversible switching capabilities [68–69], but also conformational [9–10,70], tautomeric [71], and charge state switching could be shown [56].

This Thematic Issue highlights recent experimental and theoretical developments in realizing and understanding physically and electronically decoupled single molecules and molecular assemblies on surfaces. Several decoupling strategies at the solid–vacuum and solid–liquid interface were explored to elucidate structural, electronic, vibronic, and chemical properties of decoupled molecular structures.

Physical decoupling by molecular design often relies on non-planar adsorbates with bulky spacer groups, which can adopt various conformations. From a theoretical point of view, finding the energetically most stable conformational structure can be challenging and costly because conventional atomistic simulations are often limited to the partial exploration of the potential energy landscape due to the complexity of the system. Recently developed structure search methods that combine machine learning with density functional theory provide the possibility of reliable structure identification of non-planar molecules, as demonstrated for the example of (1*S*)-camphor on Cu(111) [72]. Such computational tools become relevant for molecules with bulky spacer groups since they are very valuable for predicting and interpreting the structural and conformational properties as well as the decoupling of such molecules on surfaces.

With an appropriate molecular design, the built-in functionality of the active part of the molecule can be preserved upon adsorption on a surface. An example of the preservation of catalytic properties is demonstrated for the redox behavior of manganese porphyrins at the solid–liquid interface. Redox reactions at the axial ligands attached to the metal center of the porphyrin were observed regardless of the type of surface (highly oriented pyrolytic graphite (HOPG) and Au surfaces were used), solvent (1-phenyloctane and *n*-tetradecane) and tip material (Pt/Ir, Au, and W), which indicates that the ligands have to be decoupled from the substrates [73].

Suitable functionalization of molecules is another concept to vary their adsorption strength on metal substrates. For instance, partial fluorination of pentacene molecules decreased the adsorption strength on strongly interacting substrates such as Cu [74] but did not result in notable effects on Ag(111) [75]. Although this decoupling concept is only practical on Cu, the fluorination significantly changed the molecular multilayer growth on Ag(111) and led to a physical decoupling with a nearly bulk crystalline structure for the fluorinated pentacene.

Two articles within this Thematic Issue discuss structural templating effects at the solid–liquid interface by systematically looking at the influence of organic decoupling layers. Reynaerts et al. [76] investigated the suitability of long-chain alkanes as physical decoupling layers from a graphite surface. The occurrence of the same polymorphs for 4-tetradecyloxybenzoic acid assemblies in the presence and absence of the long-chain alkane buffer layer indicated that the influence of the substrate could not solely explain the self-assembled structures. However, the alkane buffer layer provided the possibility to monitor the STM-induced nucleation, growth, and ripening of self-assembled monolayers in a more controlled fashion. Söngen et al. [77] provide insight into the interaction of organic molecules with bulk insulators by discussing the adsorption of ethanol on both calcite and magnesite using three-dimensional AFM experiments. Although molecules adsorbed on bulk insulators are electronically decoupled, molecular self-assemblies can experience a substrate templating effect due to the presence of heterogeneous adsorption sites. Therefore, Söngen et al. [77] found on bulk calcite and magnesite that the first ethanol layer arranges in a laterally ordered way due to ionic interactions, where ethanol adopts well-defined adsorption positions on the carbonate surface. In contrast, the following layers lack this order as they reside on ethanol layers. Hence, they experience a physical decoupling due to the changing chemical environment.

Next, we outline articles that use 2D materials and ultrathin dielectric layers as decoupling layers. While on the one hand, molecular functionalization is a powerful approach to tune the electronic and optical properties of 2D materials, in particular for many practical applications [78], 2D materials, on the other hand, offer an alternative way for decoupling molecular structures from metal substrates [24]. 2D van der Waals materials are generally inert and therefore, are potentially well suited for physical decoupling of molecular structures. However, moiré patterns present due to the lattice mismatch between 2D material and its substrate might serve as structural templates for molecular adsorption and self-assembly [79–82]. The electronic decoupling depends on the electronic properties of the 2D materials as they can be insulators, semiconductors, semimetals, or metals [83].

Rothe et al. [84] demonstrated that semimetallic graphene is an appropriate buffer layer for the physical and chemical decoupling of rubrene from Pt(111). The strong molecule–surface interaction on Pt(111) is expressed by hit-and-stick adsorption due to a substantial diffusion barrier. In contrast, on graphene/Pt(111) the growth of molecular domains is facilitated. Electronically, the width of the highest occupied molecular orbital (HOMO) resonance is reduced by a factor of ten on graphene/Pt(111) compared to bare Pt(111) due to a reduction of the molecule–surface hybridization. The significantly reduced resonance width allowed for resolving vibronic states in both frontier orbitals on graphene/Pt(111) by STS.

The semiconducting 2D material MoS_2_ may act as a decoupling layer for molecules from the underlying metal substrate if the molecular resonances lie within the MoS_2_ bandgap. Hence, Yousofnejad et al. [85] found using MoS_2_ on Ag(111) as substrate that the HOMO of tetracyanoquinodimethane (TNCQ) is not decoupled because it is located in the MoS_2_ valence band, while the lowest unoccupied molecular orbital narrows but still suffers from lifetime broadening because it is situated at the conduction band onset of MoS_2_. Despite this, the vibronic states of the transiently negatively charged TCNQ could be resolved by STS.

*h*BN is an insulator and has therefore been widely used to decouple organic molecules from metal substrates. Three articles within this Thematic Issue successfully employed *h*BN to investigate the pristine properties of particular molecules. Schaal et al. [86] showed that *h*BN on Ni(111) electronically decoupled tetraphenyldibenzoperiflanthene such that the molecular vibronic progression was observable by in situ differential reflectance spectroscopy, which is otherwise only achieved for multilayers on the bare Ni. On *h*BN/Cu(111), Zimmermann et al. [87] could visualize the molecular orbitals of pyrene derivatives by STM at the submolecular level, while Brülke et al. [88] measured the fluorescence of monolayer perylenetetracarboxylic dianhydride (PTCDA), which is quenched on bare Cu(111) and would require three molecular decoupling layers to be probed on Cu(111). In all three studies using *h*BN, ordered molecular films were observed. The decoupling even allowed for the formation of complex self-assemblies such as kagome lattices by tuning the number and position of the substituents of the pyrenes derivatives [87].

Metal oxide thin films on top of metal substrates are another interesting class of ultrathin interfacial layers to decouple organic molecules and to enable the study of their electronic properties without the contribution of the underlying metal substrate. Hurdax et al. reported that both charged and neutral species of sexiphenyl can co-exist on thin dielectric MgO films on Ag(100) [89]. Due to the changed work function of the substrate, charging of the adsorbates is enabled by electron tunneling. The charge transfer strongly influences the molecular conformation by planarizing the carbon backbone as well as the self-assembly. Hurdax et al. [89] suggested that work function measurements before and after the adsorption of molecules should give insight into the electronic and physical decoupling. It should be noted that metal oxide thin films feature heterogeneous adsorption sites, which can lead to the anchoring of organic molecules having specific functional groups. Hence, the interplay of the potential energy landscape of the substrate and the intermolecular interactions steers the self-assembly in such systems. Xiang et al. [90] studied these aspects for the self-assembly of porphyrin derivatives on cobalt oxide films on top of Ir(100). While the unfunctionalized diphenylporphyrin self-assembled on the bilayer film but not on the two-bilayer film, the opposite observation was made for cyanotetraphenylporphyrin.

Physical decoupling of molecules from a semiconducting substrate is discussed for the example of both insulating CaF_2_ thin films on Si(111) [91] and hydrogen passivation of Ge(001) surfaces [92]. In the first case, three scenarios were compared: PTCDA on Si, on a thin CaF_2_, and on a thicker CaF_2_ layer. While isolated PTCDA molecules were pinned to defects on Si and also on the thin CaF_2_ layer, PTCDA was physically decoupled via the thicker CaF_2_ films and self-assembled into small islands. For FePc on H-passivated Ge(001), efficient physical decoupling facilitated the growth of large islands with upright oriented molecules, similar to their arrangement in molecular crystals.

In summary, the articles collected in this Thematic Issue highlight recent experimental and theoretical developments in the atomic- and molecular-scale understanding of physically and electronically decoupled single molecules and molecular assemblies on surfaces. Over the last decade, significant progress in this field led to a manifold of decoupling strategies for single molecules and surface-supported molecular architectures. Decoupling strategies are highly relevant to preserve the intrinsic structural, electronic, and optical properties of the molecules for performing insightful fundamental surface-science-based studies on them and, thus, play a crucial role in designing new molecule-based electronic and optoelectronic devices.

Sabine Maier and Meike Stöhr

Erlangen and Groningen, July 2021
